# The Book World of Medicine and Science

**Published:** 1896-10-10

**Authors:** 


### The Book World of Medicine and Science.

Twentieth-Century Practice: An International Encyclopædia of Modern Medical Science. Edited by Thomas L. Stedman, M.D. Volume v., "Diseases of the Skin." (London: Sampson Low, Marston and Co., 1896.

This is a complete treatise on diseases of the skin and its appendages, and we may at once say that the contributors have by their excellent articles produced a volume of great value. It is perhaps inherent in a work written by many authors that the classification of disease should fall into abeyance, and in regard to diseases of the skin it is perhaps as well that no attempt should be made either to add to existing classifications, or to criticise those that have already been propounded. At any rate, in the volume before us, after a very good and well-illustrated article on the anatomy of the skin and its appendages, the authors plunge *in medias res*, beginning by a hundred pages of well-illustrated description of the parasitic diseases by Dr. Bulkley, of New York. Under the head of epilation it is worth noting that, if the hairs are half an inch or so long, a large number may be removed at once by means of a dull table-knife so held in the hand that the hairs can be seized between its blade and the thumb. The formula is also given for epilation, sticks of sealing wax melting at a low temperature, by heating which and affixing them to the affected spot, quantities of diseased hairs may be torn out when time has been given for them to become cool. There is a long and, on the whole, a fairly satisfactory article on eczema by Dr. Hyde, of Chicago, which, however, is rather spoiled by subdivision into many varieties, especially as these varieties hardly correspond with those into which the portion devoted to treatment is divided. Squamous affections are treated by Dr. Radcliffe Crocker, of London. In regard to the moot point as to the dependence of psoriasis on a microbe or on some neuro-vascular change, Dr. Crocker says that a microbe *ab extra* will not account for the clinical facts of symmetry, and in many cases the simultaneous or rapid development of a large number of lesions on both sides of the body, although it is quite conceivable that in consequence of certain neuro-vascular changes in the skin a secondary invasion of microbes might occur on the parts so affected. Treatment by thyroid extract is spoken of with approval in certain cases, but the limits within which this drug is useful are shown to be much narrower than was at first supposed. If no effect is produced within a month it may be considered to have failed. In an interesting and well-illustrated article Professor Kaposi, of Vienna, gives an account of xeroderma pigmentosum. We need not go further in the description of this volume than to say that it has been written and edited with great care, and that it is a reliable and essentially modern guide to the diagnosis and treatment of the troublesome affections with which it deals.

The Modern Treatment of Stone in the Bladder by Litholapaxy. By P. J. Freyer, M.A., M.D., M.Ch. (London: Baillière, Tindall, and Cox. Second Edition. 1896. Price 5s.)

The opportunities which occur in India for studying calculous diseases of the urinary organs appear enormous, as may be judged from the fact which we find stated in this book that during the four years 1891-94 the operation of litholapaxy was performed 7,694 times in the Government hospitals in the North-West Provinces, Punjab, and Bombay. That so large a series of operations should be performed in patients of all ages by various surgeons with a resulting mortality of only 3·45 per cent is sufficient excuse for, nay almost demands, the publication of a complete sketch of the operation, its instruments, its methods, and its difficulties by one who has gained his experience in so large a field. There can be no doubt that for the purposes of the ordinary reader, who reads for the sake of gaining professional knowledge, this book might well have been compressed by the omission of a great deal which is historical and controversial. The treatment which the late Professor Bigelow and his operation received at the hands of Sir Henry Thompson is a matter of interest no doubt to certain people, and for the sake of historical accuracy it is desirable that Bigelow's great innovation should be duly recognised, but to the ordinary reader the constant intrusion of this topic is a little tiresome. The book, however, contains a very good and useful account of the instruments employed in litholapaxy, of the operation itself, and of the difficulties and complications which are apt to be met with, together with illustrative cases bearing on the various points raised. In regard to his experience, Dr. Freyer gives his number of cases of litholapaxy at 610, in 599 individuals ranging from 1½ to 96 years of age, and his deaths at 11, or a mortality of 1·80 per cent., being about 2 per cent. in adults and 1·17 per cent. in children. To operating surgeons perhaps the most interesting part of the book is that which refers to the performance of litholapaxy in male children. Both in regard to children and adults great stress is laid on the importance of the lithotrite being of the completely fenestrated pattern, but it is justly enough said, "When the urethra is very narrow or the stone large, the operation is a difficult one. In any case, litholapaxy in male children is a much more delicate one than in the adult."

Aids to Obstetrics. By Samuel Hall, B.A., M.B. Fifth Edition. Students' Aid Series. (London: Baillière, Tindall, and Cox. 140 pages. 1896.)

This number of the Students' Aid Series is so well known to all who have passed through the difficulties of a midwifery examination that comment or criticism is almost unnecessary. The present, or fifth edition, differs but slightly from the others which have preceded it; it has, however, been thoroughly revised and a few corrections made, and the section which deals with symphysiotomy has been entirely rewritten, in view of the importance which is attached to what may be regarded rather as a German and French method than an English one. With regard to it, Hall remarks, "Recently this operation has been reintroduced; but at present our experience is too small to say whether or not it will hold a permanent place among recognised obstetric operations." It is, however, a subject on which students are now often examined, and hence the value of a concise and practical account such as has been given in the new edition. In all other sections the details are accurate and harmonise with what is given in our leading text-books.

---

Messrs. Wright and Co., of Bristol, are about to issue a new edition of Dr. Robert Saundby's books on Bright's disease and diabetes, under the title of "Lectures on Renal and Urinary Diseases," which makes the work a complete book of reference, plates and much new matter having been added.

